# Altered Processing of Social Emotions in Individuals With Autistic Traits

**DOI:** 10.3389/fpsyg.2022.746192

**Published:** 2022-03-03

**Authors:** Di Yang, Hengheng Tao, Hongxin Ge, Zuoshan Li, Yuanyan Hu, Jing Meng

**Affiliations:** ^1^Key Laboratory of Applied Psychology, Chongqing Normal University, Chongqing, China; ^2^School of Education, Chongqing Normal University, Chongqing, China; ^3^Key Laboratory of Emotion and Mental Health, Chongqing University of Arts and Sciences, Chongqing, China

**Keywords:** autism, autistic traits, social emotion, perception, event-related potentials

## Abstract

Social impairment is a defining phenotypic feature of autism. The present study investigated whether individuals with autistic traits exhibit altered perceptions of social emotions. Two groups of participants (High-AQ and Low-AQ) were recruited based on their scores on the autism-spectrum quotient (AQ). Their behavioral responses and event-related potentials (ERPs) elicited by social and non-social stimuli with positive, negative, and neutral emotional valence were compared in two experiments. In Experiment 1, participants were instructed to view social-emotional and non-social emotional pictures. In Experiment 2, participants were instructed to listen to social-emotional and non-social emotional audio recordings. More negative emotional reactions and smaller amplitudes of late ERP components (the late positive potential in Experiment 1 and the late negative component in Experiment 2) were found in the High-AQ group than in the Low-AQ group in response to the social-negative stimuli. In addition, amplitudes of these late ERP components in both experiments elicited in response to social-negative stimuli were correlated with the AQ scores of the High-AQ group. These results suggest that individuals with autistic traits have altered emotional processing of social-negative emotions.

## Introduction

Autism spectrum disorder (ASD) is a neurodevelopmental disorder that affects 1–2% of children globally. ASD is commonly characterized by functional deficits in social behavior, difficulties in social communication, and restricted and repetitive interests (American Psychiatric Association, 2013). Such social impairment is a defining phenotypic feature of ASD (Ogawa et al., 2019; Liu et al., 2020). Previous studies of individuals with ASD have found diminished motivation to engage in social activities (Tsurugizawa et al., 2020), deficits in reciprocal social communication (De Crescenzo et al., 2019), and deficits in the ability to understand the beliefs and intentions of others (Chevallier et al., 2012; Ruta et al., 2017). It has been suggested that the core social impairment of ASD may involve impaired social motivation (Sumiya et al., 2020). This “social motivational theory” of ASD has suggested that a lack of reward feelings from social stimuli (i.e., social motivation deficit or social anhedonia) can substantially lead individuals with ASD to demonstrate a generally reduced preference for, and orientation toward, social stimuli (McPartland et al., 2012; Ruta et al., 2017) with a subsequent loss of social learning opportunities (Ruta et al., 2017; Li et al., 2019).

The autism-spectrum quotient (AQ) (Baron-Cohen et al., 2001) has been widely adopted as a way to evaluate autistic traits. It can be used to measure the core autism-related deficits in both ASD individuals and typically developing individuals. Individuals with ASD generally score at the extreme end of the AQ distribution in the general population (Dubey et al., 2015). Prior studies have found that quantifiable autistic traits are continuously distributed in typically developing individuals (Lazar et al., 2014; Sindermann et al., 2019) and that higher scores on the AQ questionnaire generally reflect a higher level of autistic traits (Ruzich et al., 2015). Individuals with high AQ scores (i.e., individuals with autistic traits or High-AQ individuals) exhibit social impairments similar to those exhibited by individuals with ASD (Poljac et al., 2012; Becker et al., 2021). Compared to individuals with low AQ scores (Low-AQ individuals), High-AQ individuals generally exhibit a reduced interest in social stimuli such as faces (Chita-Tegmark, 2016; Fogelson et al., 2019), eye gaze (Kliemann et al., 2010), social voices (Lepistö et al., 2005; Honisch et al., 2021), and social scenes (Chawarska et al., 2013; Chita-Tegmark, 2016; Tang J. S. Y. et al., 2019). These studies show that High-AQ individuals have a specific impairment in the processing of social stimuli.

Although several studies have shown that the perception of social stimuli is altered in individuals with ASD or autistic traits (Poljac et al., 2012; Chawarska et al., 2013; Robertson and Baron-Cohen, 2017; Tang J. S. Y. et al., 2019; Becker et al., 2021) these studies only used neutral social stimuli. Mental processing in individuals with ASD (Alaerts et al., 2013) or autistic traits (Azuma et al., 2015; Kerr-Gaffney et al., 2020) has been found to differ depending on the emotional valence of the stimulus, in that such individuals exhibit worse recognition of stimuli with negative rather than positive emotional valence. However, very little is known of the cognitive and neural mechanisms involved in the processing of social-emotional stimuli – a process that is fundamental to human communication (De Stefani and De Marco, 2019) – in individuals with autistic traits.

Event-related potentials (ERPs) can be used to measure the neurophysiological mechanisms underlying the processing of emotional stimuli. For example, as early components of ERP in emotion processing, N1 in both the visual modality (Xia et al., 2017; Zhao et al., 2020b) and the auditory modality (Tumber et al., 2014) reflect early attention to emotional stimuli. Larger N1 amplitudes have been observed in response to emotional pictures (Cheng et al., 2017; Tang W. et al., 2019) and emotional audio recordings (Tumber et al., 2014) as compared to neutral stimuli. In addition, the late positive potential (LPP) in the visual modality and the late negative component (LNC) in the auditory modality are late components of ERP in emotion processing that are thought to be an indicator of the evaluation of emotional information (Calbi et al., 2019; Zubair et al., 2020).

The present study aimed to investigate whether individuals with autistic traits would exhibit atypical perception of social-emotional stimuli in both the visual and auditory modalities, as measured using ERPs. As suggested by the social motivational theory of ASD (Chevallier et al., 2012), we hypothesized that individuals with autistic traits would display a similar alteration of their processing of social-emotional stimuli. In addition, as individuals with autistic traits exhibit worse recognition of negative emotional stimuli (Kerr-Gaffney et al., 2020; Meng et al., 2020), the present study hypothesized that altered perception of social-emotional stimuli in such individuals would mainly be exhibited in regard to social-negative emotions.

## Experiment 1

### Materials and Methods

#### Participants

A total of 2,592 undergraduate students aged from 18 to 23 years (Mean = 22.88 years, SD = 1.27 years) from the Chongqing Normal University were recruited to complete the Mandarin Version of the AQ questionnaire (Baron-Cohen et al., 2001; Lehnhardt et al., 2013) to estimate the magnitude of their autistic traits.

Then, two subsets of participants, those exhibiting the top 10% and bottom 10% of AQ scores (Meng et al., 2019, 2020) from the total of 2,592 undergraduate students were randomly selected and divided into High-AQ (*n* = 30) and Low-AQ (*n* = 30) groups. The detailed demographic characteristics of the participants in the High-AQ and Low-AQ groups are listed in Table 1. All participants had normal or corrected-to-normal vision and reported no history of neurological or psychiatric disorders. Written informed consent was provided by all participants prior to participation in the experiment in accordance with the Declaration of Helsinki, and the procedures of Experiment 1 were approved by the Chongqing Normal University Research Ethics Committee. The procedures were performed in accordance with ethical guidelines and regulations.

**TABLE 1:** Demographic characteristics of the High-AQ and Low-AQ groups in Experiment 1.

	High-AQ	Low-AQ	Statistics
Gender (F/M)	15/15	15/15	
Age (years) (M ± SD)	21.03 ± 1.88	20.70 ± 1.12	*t*_(58)_ = 0.833; *p* = 0.408
AQ scores (M ± SD)	26.48 ± 4.88	12.91 ± 1.97	*t*_(58)_ = 14.027; *p* < 0.001

*AQ, autism-spectrum quotient.*

*Statistical results were obtained using independent sample t-tests between the High-AQ and Low-AQ groups.*

#### Stimuli

**FIGURE 1:**
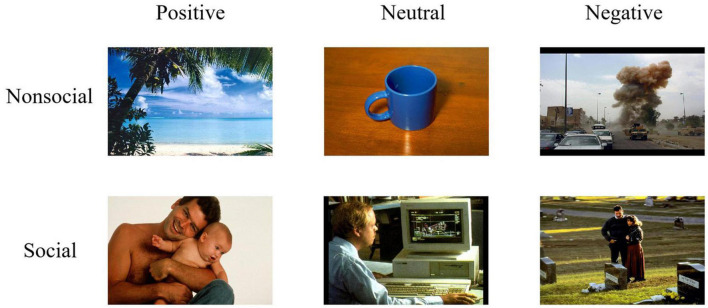
Examples of pictures used in Experiment 1. Examples of nonsocial (top panel) and social (bottom panel) pictures with positive (left column), neutral (middle column), and negative (right column) emotional valence. Pictures were selected from the International Affective Picture System (IAPS) (Lang et al., 2005) (Reproduced with permission from Di Yang), and the Chinese Affective Picture System (CAPS) (Reproduced with permission from Di Yang and Yuanyan Hu).

A total set of 144 digital pictures with negative, neutral, or positive emotional valence were selected from the International Affective Picture System (IAPS) (Lang and Bradley, 2007) and the Chinese Affective Picture System (CAPS) (Lu et al., 2005). These pictures also have either social or non-social dimensions. Social emotions rely on the presence of human forms interacting in cognitively complex ways involving language, meaning, and social intentionality to activate the emotion, whereas non-social emotions have nothing to do with social interaction and are not caused by other people (Silk and House, 2011; Tam, 2012; Mendelsohn et al., 2018). Based on previous research on the selection of social-emotional and non-social emotional stimuli (Silvers et al., 2012; Vrtička et al., 2013), our study selected 72 social pictures depicting two people or parts of people interacting or engaging in a social relationship, and 72 non-social pictures containing no images of people. These pictures were further classified into six types: social-neutral, non-social neutral, social-positive, non-social positive, social-negative, and non-social negative pictures (for examples, see Figure 1). The size of all selected pictures is 413 × 311 pixels, 10.5 cm × 7.9 cm.

Forty-three undergraduate students (21 females) assessed emotional valence (1 = *extremely negative*, 5 = *neutral*, 9 = *extremely positive*) and arousal (1 = *low arousal*, 9 = *high arousal*) produced by the selected pictures using 9-point Likert scales. In addition, they were instructed to judge whether the picture depicted social content (1 = *social* or 2 = *non-social*). The detailed descriptive statistics are summarized in Supplementary Tables 1, 2. According to the results of the assessment, social and non-social pictures were matched in emotional valence and arousal in each emotional picture.

#### Experimental Procedure

**FIGURE 2:**
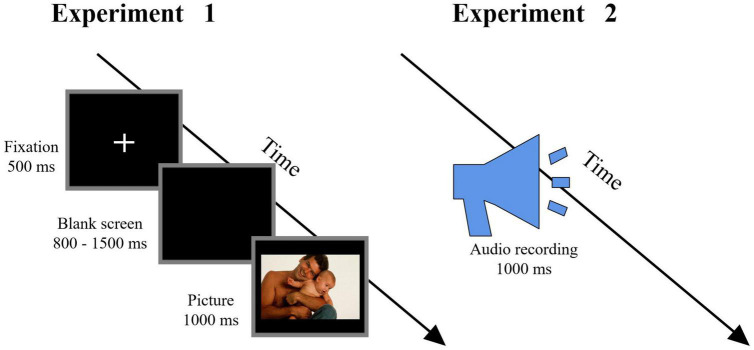
Flowchart describing the experimental design. Left column: Procedure of Experiment 1, in which participants were instructed to passively view the pictures. Right column: Procedure of Experiment 2, in which participants were instructed to passively listen to the audio recordings (Reproduced with permission from Di Yang and Yuanyan Hu).

Participants were seated in a quiet room at a comfortable temperature. As in previous studies (Hsu et al., 2020; Zhou et al., 2020), an implicit processing (passive viewing) paradigm was employed. As shown in Figure 2 (left column), at the start of a trial, a fixation cross was presented for a duration of 500 ms. After 800–1,500 ms, a picture was presented for 1,000 ms, and participants were asked to view the picture passively with attention. The order of the pictures’ presentation was randomized. The experimental procedure was programmed using the E-Prime 3.0 software (Psychology Software Tools, PA, United States). Electroencephalography (EEG) data were recorded simultaneously. The entire experimental procedure consisted of three blocks, each containing 140 trials and with an inter-trial interval of 500 ms.

After the EEG recording session, the participants were instructed to respond as accurately and quickly as possible by pressing a specific key (“1,” “2,” or “3”) to judge the emotional valence (positive, neutral, or negative) of the picture. Key-pressing was counterbalanced across participants to control for order effects. After judging the emotional valence, participants were instructed to rate their subjective emotional reactions (1 = *very unhappy*, 5 = *neutral*, 9 = *very happy*) to each picture, based on a 9-point Likert scale.

#### Electroencephalography Recording

Electroencephalography data were recorded from 64 scalp sites, using tin electrodes mounted on an actiCHamp system (Brain Vision LLC, Morrisville, NC, United States; pass band: 0.01–100 Hz; sampling rate: 1,000 Hz). The electrode at the right mastoid was used as a recording reference, and that on the medial frontal aspect was used as the ground electrode. All electrode impedances remained below 5 kΩ.

#### Electroencephalography Data Analysis

Electroencephalography data were pre-processed and analyzed via MATLAB R2014a (MathWorks, United States) and the EEGLAB v13.6.5b toolbox (Delorme and Makeig, 2004). Continuous EEG signals were band-passed filtered at 0.01–40 Hz. Time windows of 200 ms before and 800 ms after the onset of stimuli were extracted from the continuous EEG, and the extracted window was baseline-corrected by the 200 ms time interval prior to stimulus onset. The EEG epochs were visually inspected and bad trials containing significant noise from gross movements were removed. Electro-oculogram (EOG) artifacts were corrected via an independent component analysis (ICA) algorithm (Jung et al., 2001). These excluded bad trials constituted 7 ± 6.7% of the total number of trials.

After confirming scalp topographies in both the single-participant and group-level ERP waveforms, as well as previous studies (Foti and Hajcak, 2008; Hajcak et al., 2010), the dominant ERP components involved in Experiment 1 were identified, including early ERP components (N1, P2, and N2) and late ERP components (P3 and LPP). Amplitudes of N1 and N2 components were measured at the frontal-central electrodes (F1, Fz, F2, FC1, FCz, and FC2) and calculated as average ERP amplitudes within N1 latency intervals of 80–120 ms and N2 latency intervals of 210–250 ms. Amplitudes of P2, P3, and LPP components were measured at the occipital electrodes (P1, Pz, P2, PO3, POz, and PO4) and calculated as the average ERP amplitudes within P2 latency intervals of 200–240 ms, P3 latency intervals of 310–350 ms, and LPP latency intervals of 400–700 ms.

#### Statistical Analysis

Sample sizes of Experiment 1 were calculated using Gpower 3 v3.1.9.2; using a repeated measures analysis of variance (ANOVA) within-between (*F* test), with a desired power of 99%, at a 1% significance level, and an effect size of 0.29 (calculated from the interactive effect of results).

Amplitudes of dominant ERP components (N1, P2, N2, P3, and LPP) and behavioral data [accuracies (ACCs), reaction times (RTs), and subjective emotional reactions] in Experiment 1 were compared using a three-way mixed-design ANOVA, with within-participant factors of “emotion” (positive, neutral, and negative) and “sociality” (social and non-social), as well as the between-participants factor of “group” (High-AQ and Low-AQ). The Mauchly test was used in repeated measures ANOVA to test sphericity. The degrees of freedom for *F*-ratios were corrected according to the Greenhouse–Geisser method (Benjamini and Hochberg, 1995). If the interactions between the three factors were significant, simple effect analysis between groups was performed for each condition and reported in the results. Other detailed interaction effects are presented in the Experiment 1 in Supplementary Results.

In addition, to investigate the relationship between neural responses and autistic traits, a Pearson correlation was calculated between participants’ AQ scores and ERP amplitudes (N1, P2, N2, P3, and LPP) in Experiment 1.

### Results

#### Behavioral Data

**FIGURE 3:**
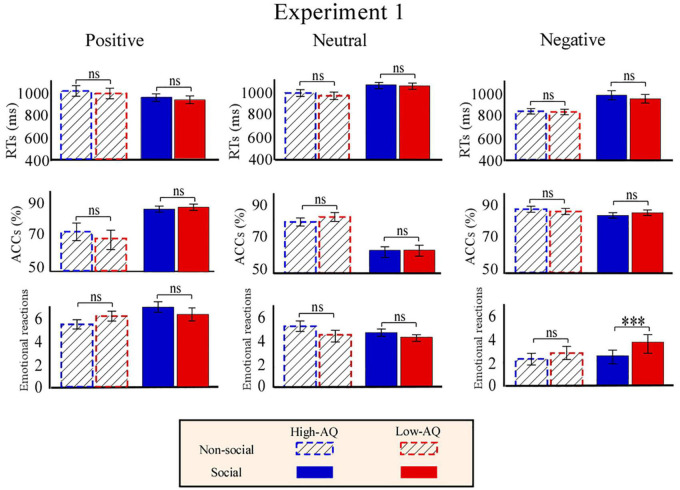
Behavioral results from Experiment 1. Bar charts show responses of High-AQ (blue) and Low-AQ (red) groups to positive **(left column)**, neutral **(middle column)**, and negative **(right column)** pictures with social (solid bar) or non-social (dotted bar) dimensions. Data of RTs, ACCs, and emotional reactions are shown in the top, middle, and bottom panels. Data in the bar charts are expressed as Mean ± SEM. ^ns^*p* > 0.05, *p < 0.05, **p < 0.01, ***p < 0.001.

Accuracies, RTs, and emotional reactions for each condition in Experiment 1 are summarized in Figure 3. RTs were modulated by the main effects of “emotion” (*F*_2,57_ = 80.86, *p* < 0.001, $\eta_{p}^{2}=0.58$) and “sociality” (*F*_1,58_ = 114.66, *p* < 0.001, $\eta_{p}^{2}=0.66$). Participants displayed longer RTs toward the neutral pictures (1,127.05 ± 28.11 ms) than toward the positive (942.80 ± 22.11 ms, *p* < 0.001) and negative (929.48 ± 21.83 ms, *p* < 0.001) pictures. No significant difference in RTs was identified between the positive and negative pictures (*p* = 0.282). In addition, participants displayed longer RTs toward the social pictures (1,035.67 ± 22.79 ms) than toward the non-social pictures (963.89 ± 21.71 ms).

Accuracies were modulated by the main effect of “emotion” (*F*_2,57_ = 45.14, *p* < 0.001, $\eta_{p}^{2}=0.44$) and “sociality” (*F*_1,58_ = 8.01, *p* = 0.006, $\eta_{p}^{2}=0.12$). Participants exhibited higher accuracy toward the negative pictures (91.5 ± 1.0%) than the positive (81.0 ± 1.9%, *p* < 0.001) and neutral (68.7 ± 2.0%, *p* < 0.001) pictures, and exhibited higher accuracy toward the positive pictures than the neutral pictures (*p* < 0.001). In addition, participants exhibited higher accuracy toward the social pictures (82.0 ± 1.0%) than the non-social pictures (78.80 ± 1.1%).

Emotional reactions were modulated by the main effect of “emotion” (*F*_2,57_ = 241.53, *p* < 0.001, $\eta_{p}^{2}=0.81$). *Post hoc* comparisons showed that participants felt more negative toward the negative pictures (2.91 ± 0.09) as compared to the positive (6.29 ± 0.15, *p* < 0.001) and neutral (4.39 ± 0.07, *p* < 0.001) pictures, and felt more negative toward the neutral pictures as compared to the positively valenced pictures (*p* < 0.001). Importantly, the participants’ emotional reactions were modulated by the interaction of “emotion” × “sociality” × “group” (*F*_2,57_ = 4.24, *p* = 0.020, $\eta_{p}^{2}=0.07$). Simple effects analysis indicated that participants’ emotional reactions to social-negative pictures were more negative for the High-AQ group than for the Low-AQ group (High-AQ group: 2.49 ± 0.78, Low-AQ group: 3.55 ± 0.74; *F*_2,57_ = 29.21, *p* < 0.001, $\eta_{p}^{2}=0.34$). However, emotional reactions were not different between groups in other conditions (*p* > 0.05 for all comparisons).

#### Event-Related Potentials Data

**FIGURE 4:**
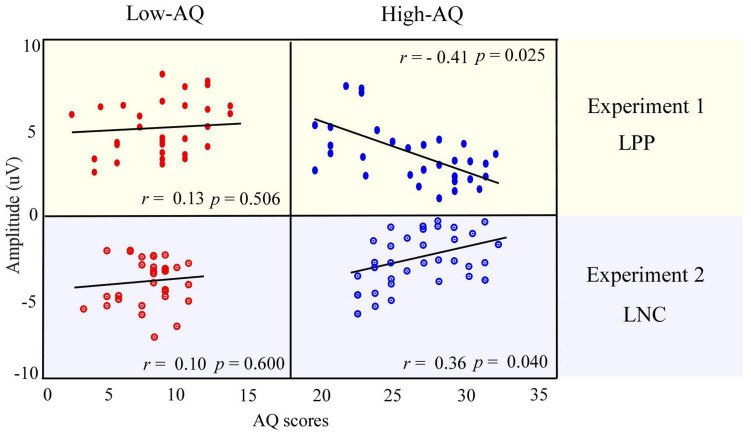
Event-related potential waveforms, scalp topography distributions, and bar charts for Experiment 1. ERP waveforms exhibited by the High-AQ (blue) and Low-AQ (red) groups in response to positively **(left column)**, neutral **(middle column)**, and negatively **(right column)** valenced pictures with social (solid) or non-social (dotted) dimensions. Electrodes used to estimate the ERP amplitudes were marked using the black squares on their respective topographic distributions. Data in the bar charts were expressed as Mean ± SEM. ^ns^*p* > 0.05, *p < 0.05, **p < 0.01, ***p < 0.001.

Amplitudes of dominant ERP components in Experiment 1 were compared by a three-way mixed-design ANOVA. The relevant results are shown in Figure 4 and Table 2.

**TABLE 2:** Summary of statistical analyses of ERP amplitudes in Experiment 1.

	N1			P2			N2			P3			LPP		
	*F*	*p*	$\eta_{p}^{2}$	*F*	*p*	$\eta_{p}^{2}$	*F*	*p*	$\eta_{p}^{2}$	*F*	*p*	$\eta_{p}^{2}$	*F*	*p*	$\eta_{p}^{2}$
Sociality	0.89	0.35	0.02	**5.09**	**0.028**	**0.08**	**12.22**	**0.001**	**0.17**	**6.49**	**0.014**	**0.10**	**4.34**	**0.042**	**0.07**
Emotion	**14.24**	<**0.001**	**0.20**	1.24	0.293	0.02	**26.05**	<**0.001**	**0.31**	**4.86**	**0.011**	**0.08**	**11.41**	<**0.001**	**0.16**
Group	0.87	0.354	0.02	0.55	0.463	0.01	0.61	0.439	0.01	0.43	0.513	0.01	1.44	0.235	0.02
Sociality × emotion	3.05	0.054	0.05	1.22	0.299	0.02	0.81	0.452	0.03	**4.31**	**0.018**	**0.07**	**6.34**	**0.003**	**0.10**
Sociality × group	1.78	0.187	0.03	2.09	0.154	0.04	0.003	0.957	<0.01	0.04	0.949	<0.01	0.17	0.685	<0.01
Emotion × group	1.22	0.298	0.02	1.32	0.271	0.02	1.10	0.335	0.02	1.26	0.288	0.02	2.35	0.103	0.04
Sociality × emotion × group	1.23	0.295	0.02	**3.96**	**0.023**	**0.06**	3.07	0.058	0.05	**3.89**	**0.026**	**0.06**	**3.15**	**0.047**	**0.05**

*Results were obtained using a three-way mixed-design ANOVA with within-participant factors of “emotion” (positive, neutral, and negative) and “sociality” (social and non-social), as well as the between-participants factor of “group” (High-AQ and Low-AQ). Significant (p < 0.05) comparisons are indicated in boldface.*

##### N1

The N1 amplitudes were significantly modulated by the main effect of “emotion” (*F*_2,57_ = 14.24, *p* < 0.001, $\eta_{p}^{2}=0.20$). Negative pictures (−2.91 ± 0.35 μV) elicited larger N1 amplitudes than did positive (−2.01 ± 0.34 μV, *p* < 0.001) and neutral (−2.25 ± 0.35 μV, *p* < 0.001) pictures. However, no significant difference was observed in N1 amplitudes elicited by the positive and neutral pictures (*p* = 0.190).

##### P2

The P2 amplitudes were significantly modulated by the main effect of “sociality” (*F*_1,58_ = 5.09, *p* = 0.028, $\eta_{p}^{2}=0.08$). Non-social pictures (7.32 ± 0.63 μV) elicited larger P2 amplitudes than social pictures (6.87 ± 0.64 μV).

##### N2

The N2 amplitudes were significantly modulated by the main effects of “sociality” (*F*_1,58_ = 12.22, *p* = 0.001, $\eta_{p}^{2}=0.17$) and “emotion” (*F*_2,57_ = 26.06, *p* < 0.001, $\eta_{p}^{2}=0.31$). Social pictures (−9.77 ± 0.71 μV) elicited larger N2 amplitudes than non-social pictures (−8.78 ± 0.63 μV). Negative pictures (−9.98 ± 0.65 μV) elicited larger N2 amplitudes than positive (−8.40 ± 0.67 μV, *p* < 0.001) and neutral pictures (−9.45 ± 0.70 μV, *p* = 0.011). In addition, neutral pictures elicited larger N2 amplitudes than positive pictures (*p* < 0.001).

##### P3

The P3 amplitudes were significantly modulated by the main effects of “sociality” (*F*_1,58_ = 6.49, *p* = 0.014, $\eta_{p}^{2}=0.10$) and “emotion” (*F*_2,57_ = 4.86, *p* = 0.011, $\eta_{p}^{2}=0.08$). Social pictures (7.91 ± 0.66 μV) elicited larger P3 amplitudes than non-social pictures (7.27 ± 0.64 μV). Positive pictures (8.07 ± 0.58 μV) elicited larger P3 amplitudes than negative (7.40 ± 0.68 μV, *p* = 0.011) and neutral (7.29 ± 0.69 μV, *p* = 0.013) pictures. However, no significant difference was observed in P3 amplitudes elicited by the negative and neutral pictures (*p* = 0.654).

##### Late Positive Potential

The LPP amplitudes were significantly modulated by the main effects of “sociality” (*F*_1,58_ = 4.34, *p* = 0.042, $\eta_{p}^{2}=0.07$) and “emotion” (*F*_2,57_ = 12.22, *p* = 0.001, $\eta_{p}^{2}=0.17$). Social pictures (6.77 ± 0.72 μV) elicited larger LPP amplitudes than non-social pictures (6.18 ± 0.69 μV). Positive pictures (7.51 ± 0.59 μV) elicited larger LPP amplitudes than negative (6.33 ± 0.76 μV, *p* = 0.003) and neutral (5.69 ± 0.78 μV, *p* < 0.001) pictures. However, no significant difference was observed in LPP amplitudes elicited by the negative and neutral pictures (*p* = 0.064).

Importantly, LPP amplitudes were modulated by the interaction of “emotion” × “sociality” × “group” (*F*_2,57_ = 3.15, *p* = 0.047, $\eta_{p}^{2}=0.05$). As shown in Figure 4, simple effects analysis indicated that, for social-negative pictures, LPP amplitudes were smaller in the High-AQ group than in the Low-AQ group (High-AQ group: 4.66 ± 1.07 μV, Low-AQ group: 7.98 ± 1.06 μV; *F*_2,57_ = 4.83, *p* = 0.032, $\eta_{p}^{2}=0.08$). However, no significant difference was observed between groups in other conditions (*p* > 0.05 for all comparisons).

#### Correlation Between Event-Related Potential Data and Autism-Spectrum Quotient Scores

**FIGURE 5:**
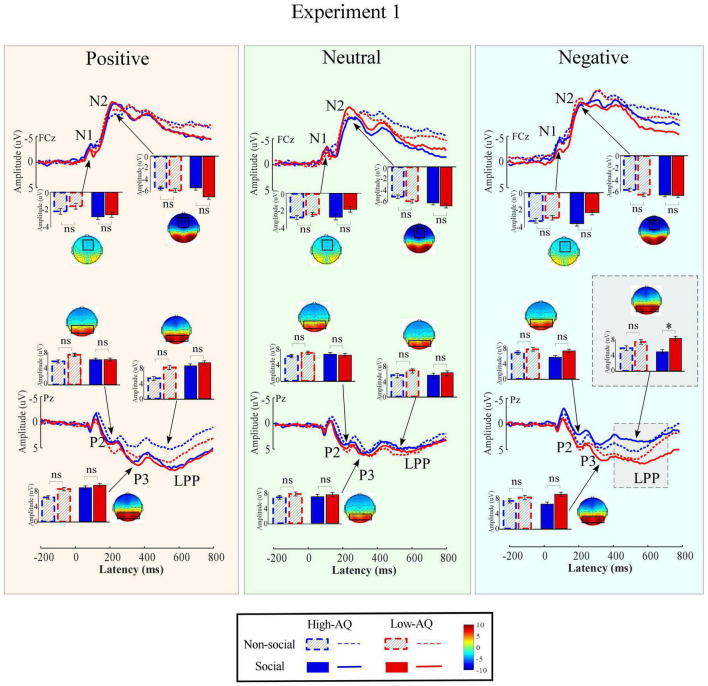
Relationship between AQ scores and ERP amplitudes. The correlation between the LPP amplitudes in response to social-negative pictures in Experiment 1 and AQ scores of the High-AQ (blue dots) and Low-AQ (red dots) groups are shown in the **top panel**. The correlation between the LNC amplitudes in response to social-negative audio recordings and AQ scores of the High-AQ (blue dots) and Low-AQ (red dots) groups in Experiment 2 are shown in the **bottom panel**. Data in the figure were calculated using the Pearson correlation coefficient.

For social-negative pictures, the LPP amplitudes were negatively correlated with the AQ scores of the High-AQ group (*r* = −0.41, *p* = 0.025), but not correlated with the AQ scores of the Low-AQ group (*r* = 0.13, *p* = 0.506). No other reliable correlation was found between AQ scores and ERP amplitudes in other conditions (*p* > 0.05 for all correlations). The correlation results are displayed in Figure 5 (top panel).

## Experiment 2

### Materials and Methods

#### Participants

A total of 2,083 undergraduate students aged from 18 to 23 years (Mean = 21.18 years, SD = 1.68 years) from the Chongqing Normal University (who did not participate in Experiment 1) were recruited to complete the Mandarin Version of the AQ questionnaire (Baron-Cohen et al., 2001; Lehnhardt et al., 2013).

Then, a subset of participants (those exhibiting the top 10% and bottom 10% of AQ scores) (Meng et al., 2019, 2020) were randomly selected and divided into High-AQ (*n* = 33) and Low-AQ (*n* = 32) groups. The detailed demographic characteristics of the High-AQ and Low-AQ groups are listed in Table 3. All the participants had normal hearing and no history of neurological or psychiatric disorders. Informed consent was given by all participants before the formal experiment in accordance with the Declaration of Helsinki, and all procedures were approved by the Chongqing Normal University Research Ethics Committee. The procedures were performed in accordance with ethical guidelines and regulations.

**TABLE 3:** Demographic characteristics of the High-AQ and Low-AQ groups in Experiment 2.

	High-AQ	Low-AQ	Statistics
Gender (F/M)	16/17	16/16	
Age (years) (M ± SD)	21.36 ± 1.78	21.00 ± 1.59	*t*_(63)_ = 0.868; *p* = 0.389
AQ scores (M ± SD)	29.72 ± 2.34	11.65 ± 2.22	*t*_(63)_ = 31.551; *p* < 0.001

*AQ, Autism Spectrum Quotient.*

*Statistical results were obtained using independent sample t-tests between the High-AQ and Low-AQ groups.*

#### Stimuli

A total of 60 negative, neutral, and positive audio recordings were selected from the Montreal Affective Voices database (Belin et al., 2008) and the Chinese Affective Voices database (Liu and Pell, 2012). These audio recordings have either social or non-social dimensions. Based on previous research on the selection of social-emotional and non-social emotional stimuli (Silvers et al., 2012; Vrtička et al., 2013), our study selected 30 social audio recordings depicting a male or female human voice voicing the vowels, such as/a/; and 30 non-social audio recordings depicting non-human sounds, such as audio recordings of musical instruments, tools, and animals. These audio recordings were then further classified into six types: social-neutral, non-social neutral, social-positive, non-social positive, social-negative, and non-social negative. These categories are consistent with the types of stimuli in Experiment 1.

All audio recordings were edited to last 1,000 ms, with a mean intensity of 70 dB (Panksepp and Bernatzky, 2002). All audio recordings were evaluated by 40 undergraduate students (20 females). They were asked to evaluate emotional valence (1 = *extremely negative*, 5 = *neutral*, 9 = *extremely positive*) and arousal (1 = *not at all aroused*, 9 = *extremely aroused*) produced by the audio recordings by using 9-point Likert scales. They were also instructed to judge whether the audio recordings involved social content (1 = *social* or 2 = *non-social*). The detailed descriptive statistics are summarized in Supplementary Tables 3, 4. According to the results of the assessment, social and non-social audio recordings were matched in emotional valence and arousal.

#### Experimental Procedure

Participants were seated in a quiet room at a comfortable temperature. As in previous studies (Hsu et al., 2020; Zhou et al., 2020), an implicit processing (passive listening) paradigm was employed and participants were asked to attentively listen to the audio recordings (Figure 2, right column). Each audio recording lasted for 1,000 ms with an inter-stimulus interval (ISI) of 1.5–2.5 s, and the order of audio recordings was randomized. The experimental procedure was programmed using the E-Prime 3.0 software (Psychology Software Tools, PA, United States). EEG data were recorded simultaneously. The whole experimental procedure consisted of two blocks, each containing 210 audio recordings.

After each EEG recording session, the participants were instructed to respond as accurately and quickly as possible by pressing a specific key (either “1,” “2,” or “3”) to judge the emotional valence of the audio recording (positive, neutral, or negative). Key-pressing was counterbalanced across participants to control for order effects. After judging the emotional valence, participants were instructed to rate their subjective emotional reactions (1 = *very unhappy*, 5 = *neutral*, 9 = *very happy*) to each audio recording, based on a 9-point Likert scale.

#### Electroencephalography Recording

Same as Experiment 1.

#### Electroencephalography Data Analysis

The EEG data pre-processing procedure of Experiment 2 is the same as that used in Experiment 1. These excluded bad trials constituted 5 ± 2.1% of the total number of trials.

After confirming scalp topographies in both single-participant and group-level ERP waveforms, and based on previous studies (Cheng et al., 2017; Meng et al., 2020), the dominant ERP components involved in Experiment 2 were identified, including early ERP components (N1 and P2) and LNC. N1 and P2 were identified as the most negative and positive deflections, respectively, at 100–300 ms after the audio recording’s onset with maximum distribution at the frontal-central electrodes. The LNC distributed in the frontal-central electrodes was a long-lasting negative wave within latency intervals of 300–700 ms after the audio recording’s onset. Amplitudes of N1 and P2 were both measured at the frontal-central electrodes (Fz, F1, F2, FCz, FC1, FC2, Cz, C1, and C2) and calculated as the average ERP amplitudes within N1 latency intervals of 100–120 ms and P2 latency intervals of 190–210 ms. Amplitudes of LNC were measured at the prefrontal electrodes (Fz, F1, F2, FCz, FC1, and FC2) and calculated as the average ERP amplitudes within latency intervals of 300–700 ms.

#### Statistical Analysis

Sample sizes of Experiment 2 were calculated using Gpower 3 v3.1.9.2 (see text footnote 1); using a repeated measures ANOVA within-between (*F* test), with a desired power of 99%, at a 1% significance level, and an effect size of 0.25 (calculated from the interactive effect of results).

In Experiment 2, amplitudes of dominant ERP components (N1, P2, and LNC) and behavioral data (ACCs, RTs, and subjective emotional reactions) were compared using three-way mixed-design ANOVA, with within-participant factors of “emotion” (positive, neutral, and negative) and “sociality” (social and non-social), as well as the between-participants factor of “group” (High-AQ and Low-AQ). If the interactions between the three factors were significant, simple effect analysis was performed between groups for each condition and reported in the results. Other detailed interaction effects are presented in the Experiment 2 in Supplementary Results.

In addition, to investigate the relationship between the neural responses and autistic traits, a Pearson correlation was calculated between participants’ AQ scores and the amplitudes of the ERP components (N1, P2, and LNC) in Experiment 2.

### Results

#### Behavioral Data

**FIGURE 6:**
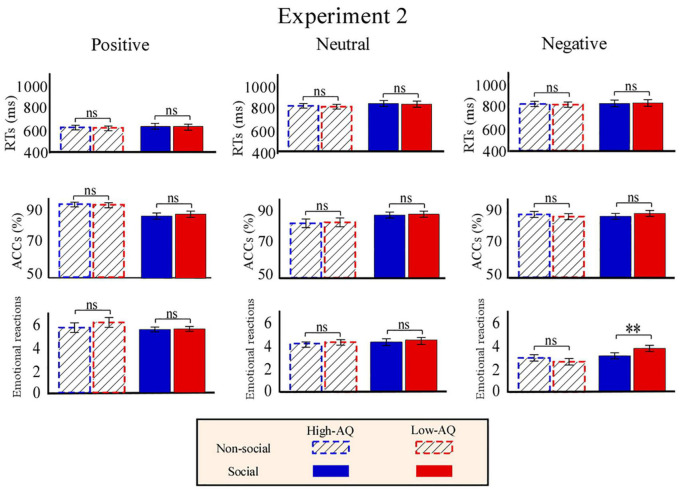
Behavioral results from Experiment 2. Bar charts show responses of High-AQ (blue) and Low-AQ (red) groups to positive **(left column)**, neutral **(middle column)**, and negative **(right column)** pictures with social (solid bar) or non-social (dotted bar) dimensions. Data of RTs, ACCs, and emotional reactions are shown in the top, middle, and bottom panels. Data in the bar charts are expressed as Mean ± SEM. ^ns^*p* > 0.05, *p < 0.05, **p < 0.01, ***p < 0.001.

As shown in Figure 6, RTs were modulated by the main effect of “sociality” (*F*_1,63_ = 11.27, *p* = 0.001, $\eta_{p}^{2}=0.15$) with participants displaying longer RTs toward the social audio recordings (633.15 ± 12.66 ms) than toward the non-social audio recordings (606.71 ± 9.67 ms).

Accuracies were modulated by the main effect of “emotion” (*F*_2,62_ = 3.19, *p* = 0.046, $\eta_{p}^{2}=0.48$). *Post hoc* comparisons showed participants displayed higher ACCs toward the positive audio recordings (87.9 ± 1.0%) than toward the negative audio recordings (81.0 ± 1.9%, *p* = 0.047).

Emotional reactions were modulated by the main effect of “sociality” (*F*_1,63_ = 5.62, *p* = 0.021, $\eta_{p}^{2}=0.08$) and “emotion” (*F*_2,62_ = 543.77, *p* < 0.001, $\eta_{p}^{2}=0.89$). Participants felt more negative toward the social audio recordings (5.05 ± 0.05) than toward the non-social audio recordings (4.89 ± 0.08). Participants felt more negative toward the negative audio recordings (3.10 ± 0.08) than toward the positive (7.03 ± 0.12, *p* < 0.001) and neutral (4.77 ± 0.05, *p* < 0.001) audio recordings, and felt more negative toward the neutral audio recordings than toward the positive audio recordings (*p* < 0.001). Importantly, emotional reactions were modulated by the interaction of “emotion” × “sociality” × “group” (*F*_2,62_ = 8.21, *p* = 0.001, $\eta_{p}^{2}=0.12$). Simple effects analysis indicated that for social-negative audio recordings, the High-AQ group felt more negative than the Low-AQ group (High-AQ: 3.14 ± 0.14, Low-AQ: 3.81 ± 0.13; *F*_2,62_ = 12.57, *p* = 0.001, $\eta_{p}^{2}=0.17$). However, there was no group difference observed in the other conditions (*p* > 0.05 for all comparisons), as shown in Figure 6.

#### Event-Related Potential Data

**FIGURE 7:**
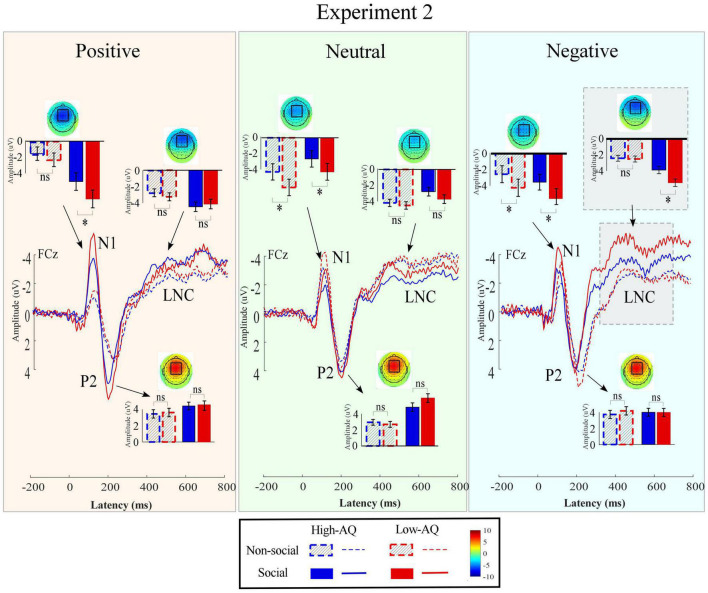
Event-related potentials waveforms, scalp topography distributions, and bar charts in Experiment 2. ERP waveforms were exhibited by the High-AQ (blue) and Low-AQ (red) groups passively listening to positively **(left column)**, neutral **(middle column)**, and negatively **(right column)** valenced audio recordings with social (solid) or non-social (dotted) dimensions. Electrodes used to estimate the mean ERP amplitudes were marked using the black squares on their respective topographic distributions. Data in the bar charts were expressed as Mean ± SEM. ^ns^*p* > 0.05, *p < 0.05, **p < 0.01, ***p < 0.001.

Amplitudes of dominant ERP components in Experiment 2 were compared by a three-way mixed-design ANOVA. The relevant results are shown in Figure 7 and Table 4.

**TABLE 4:** Summary of statistical analyses of ERP amplitudes in Experiment 2.

	N1			P2			LNC		
	*F*	*p*	$\eta_{p}^{2}$	*F*	*p*	$\eta_{p}^{2}$	*F*	*p*	$\eta_{p}^{2}$
Sociality	**37.21**	<**0.001**	**0.37**	**29.95**	<**0.001**	**0.32**	**13.60**	<**0.001**	**0.18**
Emotion	0.58	0.552	0.01	0.19	0.822	0.003	0.54	0.570	0.01
Group	**7.92**	**0.007**	**0.11**	0.21	0.650	0.003	0.93	0.340	0.01
Sociality × emotion	**108.13**	<**0.001**	**0.63**	**22.11**	<**0.001**	**0.26**	**31.41**	<**0.001**	**0.33**
Sociality × group	1.44	0.235	0.02	0.46	0.501	0.01	0.87	0.355	0.01
Emotion × group	0.42	0.646	0.01	0.33	0.711	0.01	1.71	0.188	0.03
Sociality × emotion × group	2.34	0.102	0.04	3.16	0.052	0.05	**3.86**	**0.025**	**0.06**

*Results were obtained using repeated-measures ANOVA with the within-participant factors of “emotion” (positive, neutral, and negative) and “sociality” (social and non-social), as well as the between-participants factor of “group” (High-AQ and Low-AQ). Significant comparisons (p < 0.05) are indicated in boldface.*

##### N1

The N1 amplitudes were modulated by the main effects of “sociality” (*F*_1,63_ = 37.21, *p* < 0.001, $\eta_{p}^{2}=0.37$) and “group” (*F*_1,63_ = 7.92, *p* = 0.007, $\eta_{p}^{2}=0.11$). Social audio recordings elicited larger N1 amplitudes than non-social audio recordings (social: −3.50 ± 0.25 μV, non-social: −2.57 ± 0.22 μV). N1 amplitudes in the High-AQ group were smaller than in the Low-AQ group (High-AQ: −2.41 ± 0.31 μV, Low-AQ: −3.66 ± 0.32 μV).

##### P2

The P2 amplitudes were modulated by the main effect of “sociality” (*F*_1,63_ = 29.95, *p* < 0.001, $\eta_{p}^{2}=0.32$) with social audio recordings eliciting larger P2 amplitudes than non-social audio recordings (social: 4.22 ± 0.30 μV, non-social: 3.14 ± 0.27 μV).

##### Late Negative Component

The LNC amplitudes were modulated by the main effect of “sociality” (*F*_1,63_ = 13.60, *p* < 0.001, $\eta_{p}^{2}=0.18$) with social audio recordings eliciting larger LNC amplitudes than non-social audio recordings (social: −2.92 ± 0.23 μV, non-social: −2.31 ± 0.18). Importantly, the LNC amplitudes were significantly modulated by the interaction effect of “emotion” × “sociality” × “group” (*F*_2,62_ = 3.86, *p* = 0.025, $\eta_{p}^{2}=0.06$). Simple effects analysis indicated that for social-negative audio recordings, LNC amplitudes in the High-AQ group were smaller than in the Low-AQ group (High-AQ group: −2.93 ± 0.34 μV, Low-AQ: −4.12 ± 0.35 μV; *F*_2,62_ = 5.77, *p* = 0.019, $\eta_{p}^{2}=0.08$). However, there was no group difference observed in the other conditions (*p* > 0.05 for all comparisons).

#### Correlation Between Event-Related Potential Data and Autism-Spectrum Quotient Scores

The LNC amplitudes elicited by social-negative audio recordings were positively correlated with the AQ scores of the High-AQ group (*r* = 0.36, *p* = 0.040) but not correlated for the Low-AQ group (*r* = 0.10, *p* = 0.600), as seen in Figure 5 (bottom panel). However, no other reliable correlations between AQ scores and ERP amplitudes were found (*p* > 0.05).

## Discussion

The present study attempted to investigate whether High-AQ individuals would exhibit altered perception of social-emotional stimuli. Results showed that the behavioral and neural responses to social-negative stimuli in both the visual and auditory modalities differed between groups. More negative emotional reactions and smaller late ERP components (LPP in Experiment 1 and LNC in Experiment 2) in response to both social-negative pictures and social-negative audio recordings were found in the High-AQ group as compared to the Low-AQ group. Thus, the present study suggests that individuals with autistic traits may exhibit altered social perception in response to social-negative stimuli.

### Experiment 1

In agreement with previous studies (Kissler and Bromberek-Dyzman, 2021), the results of Experiment 1 showed the N1 amplitudes elicited by the positive and negative pictures were larger than those elicited by the neutral pictures. Since the N1 component represents the early sensory and attention processing of visual information (Huang et al., 2017; Xia et al., 2017; Oeur and Margulies, 2020; Zhao et al., 2020a), the present results suggest that early sensory and attention processing resources were activated by emotional pictures in Experiment 1. In addition, in agreement with previous studies showing that social stimuli elicited larger amplitudes relative to non-social stimuli in the N2 time window in the visual modality (Fishman et al., 2011), the present study showed larger N2 amplitudes to be elicited by social pictures rather than non-social pictures. The N2 amplitudes evoked by visual stimuli have been suggested to be an effective index of attention (Ort et al., 2019), with a higher level of attention to stimuli inducing a larger N2 amplitude in the visual modality (Orlandi and Proverbio, 2019). Thus, our findings regarding N2 in Experiment 1 suggest that social pictures captured more attention than non-social pictures.

Also in agreement with previous studies (Calbi et al., 2019) LPP amplitudes in Experiment 1 were modulated by “emotion,” with positive pictures eliciting larger amplitudes relative to neutral pictures. As LPP over the posterior parietal cortical area is relevant to the conscious evaluation of emotional stimuli (Leventon et al., 2014), and the LPP amplitudes are positively correlated to the valence of the emotional stimuli (Suo et al., 2017), it appears, based on the present study design, that the mental processing resources of emotional valence evaluation were recruited under passive viewing conditions, and that more mental resources were involved in the response to positive pictures than to neutral pictures.

More importantly, LPP amplitudes were modulated by the interaction of “sociality,” “emotion,” and “group.” That is to say, for the social-negative pictures, LPP amplitudes in the High-AQ group were lower than those in the Low-AQ group, whereas no group difference was found for other kinds of pictures (i.e., social-neutral, non-social neutral, social-positive, non-social positive, and non-social negative). In addition, LPP amplitudes in response to social-negative pictures were correlated with the AQ scores of the participants in the High-AQ group, such that the higher the AQ scores of the participants, the smaller the LPP amplitudes elicited in response to social-negative pictures. This suggests that the LPP amplitudes evoked by social-negative pictures were sensitive to the degree of autistic traits in the High-AQ group. Thus, these results suggest that for the High-AQ group, fewer mental processing resources for emotional valence evaluation were involved in the response to social-negative pictures. This supports our hypothesis that altered processing of social-negative stimuli in the visual modality can be found in individuals with autistic traits.

### Experiment 2

In agreement with prior studies (Solberg Økland et al., 2019; Hosaka et al., 2021), the N1 amplitudes elicited by the social audio recordings (human voices) in Experiment 2 were larger than those elicited by the non-social audio recordings (non-human sounds). Since the N1 component in the auditory modality is an index of early auditory processing (Zhao et al., 2016), and focused auditory attention could result in larger N1 amplitudes in response to audio recordings (Tumber et al., 2014), our findings suggest that social audio recordings capture more attention than non-social audio recordings. In addition, our results showed that the N1 amplitudes were modulated by the main effect of “group,” in that the N1 amplitudes of the High-AQ group were smaller than those of the Low-AQ group, which is in agreement with prior studies of individuals with autistic traits (Meng et al., 2019) and ASD (Ruta et al., 2017). This suggests that the High-AQ group’s attention to audio recordings in the implicit processing (passive listening) paradigm was less than that of the Low-AQ group.

In agreement with prior ERP studies (Cheng et al., 2017; Keuper et al., 2018), the LNC amplitudes in Experiment 2 were modulated by “emotion,” with positive and negative audio recordings eliciting larger amplitudes in the LNC time window than neutral audio recordings. As LNC over the posterior parietal cortical area is relevant to the conscious cognition of emotional stimuli (Leventon et al., 2014), and LNC amplitudes are positively correlated with the evaluation of the subjective emotional valence of emotional stimuli (Suo et al., 2017), it appears that more mental processing resources were recruited for evaluation of positive and negative audio recordings as compared to neutral audio recordings.

Importantly, LNC amplitudes were modulated by the interaction between “sociality,” “emotion,” and “group.” For the social-negative audio recordings, LNC amplitudes in the High-AQ group were lower than those in the Low-AQ group, whereas no difference was found between the two groups for other kinds of audio recordings. Thus, our results suggest that the High-AQ group exhibited impaired mental processing for evaluation of social-negative audio recordings. In addition, LNC amplitudes in response to social-negative audio recordings were correlated with the AQ scores of participants in the High-AQ group: the higher the AQ score, the smaller the LNC amplitudes in response to social-negative audio recordings. These results suggest that the LNC amplitudes elicited in response to social-negative audio recordings were sensitive to the magnitude of autistic traits, supporting our hypothesis that altered perception of social-negative stimuli in the auditory modality can be found in individuals with autistic traits.

### Altered Social-Emotional Processing by Individuals With Autistic Traits

A key result of this study is the fact that both Experiment 1 and Experiment 2 found significant behavioral differences between the High-AQ and Low-AQ groups in response to social-negative stimuli. That is to say, in both experiments, the High-AQ group exhibited more negative emotional reactions to the social-negative stimuli than did the Low-AQ group. In addition, ERP results showed that LPP amplitudes elicited in response to social-negative pictures (Experiment 1) and LNC amplitudes elicited in response to social-negative audio recordings (Experiment 2) were lower in the High-AQ group than in the Low-AQ group. Both LPP and LNC amplitudes were correlated with the AQ scores of the High-AQ group only when they were elicited by social-negative stimuli.

These results support our hypothesis that individuals with autistic traits have altered processing of social-negative stimuli. Prior studies have suggested that social emotions have greater emotional significance than non-social emotions (Tam, 2012), which could activate individuals’ intrinsic motivation to respond to social emotions (Gourisankar et al., 2018). According to the social motivational theory of ASD (Scheggi et al., 2020), one possible explanation for these findings could be that individuals with autistic traits lack the intrinsic motivation to process socially significant emotional stimuli. In addition, autistic individuals exhibit enhanced perception, attention, and memory capabilities, which may cause their experience of the world to become too intense and even aversive (Markram et al., 2007), causing many of the key autistic symptoms, such as disorders of social interaction (Stavropoulos and Carver, 2018) and perception (Cascio et al., 2015). Therefore, the atypical perception of social-negative emotions by individuals with autistic traits may be the result of their overly intense and avoidant processing of social-negative stimuli in comparison to other types of social-emotional or non-social emotional stimuli. The social-negative stimuli may make individuals with autistic traits experience emotions that are too intense and thus contribute to a decline in their intrinsic motivation.

Despite these potential implications, several limitations of the present study should be noted. First, although the influence of autistic traits on reactions to social or non-social emotional stimuli was assessed in an experimental setting, whether and how these reactions are related to real-world behavior requires further investigation. Second, the present study used a passive listening/viewing paradigm. Filler trials were not used to evaluate whether participants focused sufficiently on the emotional stimuli, and thus further investigations should take this into account.

## Conclusion

This study investigated the influence of autistic traits on the perception of social-emotional stimuli. ERPs were used to measure the neural reactions that were elicited by social-emotional stimuli in both the High-AQ and Low-AQ groups. More negative emotional reactions and smaller late ERP component amplitudes (LPP of pictures and LNC of audio recordings) were found in the High-AQ group than in the Low-AQ group in response to the social-negative stimuli. In addition, LPP or LNC amplitudes in response to social-negative stimuli were correlated with the AQ scores of the High-AQ group. These results suggest that individuals with autistic traits exhibit altered behavioral and neural processing of social-negative emotional stimuli.

## Data Availability Statement

The datasets presented in this study can be found in online repositories. The names of the repository/repositories and accession number(s) can be found in the article/Supplementary Material.

## Ethics Statement

The studies involving human participants were reviewed and approved by the Chongqing Normal University Research Ethics Committee. The patients/participants provided their written informed consent to participate in this study.

## Author Contributions

DY: conceptualization, methodology, software, data curation, and writing—original draft preparation. HT: methodology, software, and writing—original draft preparation. HG: data curation and writing—original draft preparation. ZL: supervision. YH: resources. JM: conceptualization, methodology, funding acquisition, and writing—reviewing and editing. All authors contributed to the article and approved the submitted version.

## Conflict of Interest

The authors declare that the research was conducted in the absence of any commercial or financial relationships that could be construed as a potential conflict of interest.

## Publisher’s Note

All claims expressed in this article are solely those of the authors and do not necessarily represent those of their affiliated organizations, or those of the publisher, the editors and the reviewers. Any product that may be evaluated in this article, or claim that may be made by its manufacturer, is not guaranteed or endorsed by the publisher.

## Abbreviations

ASD: autism spectrum disorder
AQ: autism-spectrum quotient
ERP: event-related potential
DSM-5: Diagnostic and Statistical Manual of Mental Disorders (5th ed.)
IAPS: International Affective Picture System
CAPS: Chinese Affective Picture System
ISI: inter-stimulus interval
ANOVA: analysis of variance
EEG: electroencephalography
ACCs: accuracies
RTs: reaction times
EOG: electro-oculogram
ICA: independent component analysis.
