# Supplementary material for: Altered Processing of Social Emotions in Individuals With Autistic Traits
Source: Front Psychol. 2022 Mar 3;13:746192. doi: 10.3389/fpsyg.2022.746192 (PMC8931733; doi:10.3389/fpsyg.2022.746192)
Supplement: Supplementary file 1 [file Data_Sheet_1.docx]

**Supplementary material**

1. **Table 1. Results of pictures evaluation data in Experiment 1 (Mean ± SEM).**

|  | **Social pictures** | | | **Nonsocial pictures** | | |
| --- | --- | --- | --- | --- | --- | --- |
|  | **Positive** | **Neutral** | **Negative** | **Positive** | **Neutral** | **Negative** |
| **Emotional Valence** | 7.35 ± 0.24 | 4.99 ± 0.47 | 2.19 ± 0.44 | 7.27 ± 0.28 | 5.11 ± 0.38 | 2.14 ± 0.27 |
| **Arousal** | 4.98 ± 0.19 | 4.83 ± 0.63 | 5.01 ± 0.43 | 4.95 ± 0.29 | 4.81 ± 1.00 | 5.04 ± 0.28 |

**Note:** Total stimulus material of pictures evaluation data (Mean ± SEM).

**S. Table 2. Summary of statistical analyses of evaluation data of pictures in Experiment 1.**

|  |  | **Emotional valence** | | **Arousal** | |
| --- | --- | --- | --- | --- | --- |
|  |  | ***t*** | ***p*** | ***t*** | ***p*** |
| **Positive** | **Social** | -1.15 | 0.258 | -0.41 | 0.684 |
|  | **Nonsocial** |  |  |  |  |
| **Neutral** | **Social** | 0.98 | 0.334 | -0.09 | 0.931 |
|  | **Nonsocial** |  |  |  |  |
| **Negative** | **Social** | -0.38 | 0.703 | 0.29 | 0.769 |
|  | **Nonsocial** |  |  |  |  |

**Note:** Total stimulus material of pictures evaluation data by *t*-test. *p*-values associated with a single sample *t*-test on the differences of emotional valence and arousal between social or nonsocial pictures*.* Stimuli were assessed by 43 participants who are not in the formal experiment.

**S. Table 3. Results of audio recordings evaluation data in Experiment 2 (Mean ± SEM).**

|  | **Social pictures** | | | **Nonsocial pictures** | | |
| --- | --- | --- | --- | --- | --- | --- |
|  | **Positive** | **Neutral** | **Negative** | **Positive** | **Neutral** | **Negative** |
| **Emotional Valence** | 6.95±0.19 | 4.62±0.16 | 2.91±0.41 | 6.80±0.24 | 4.72±0.41 | 3.19±0.39 |
| **Arousal** | 6.46±0.31 | 4.01±0.14 | 5.02±0.44 | 6.48±0.86 | 4.07±0.14 | 5.13±0.41 |

**Note:** Total stimulus material of sounds evaluation data (Mean ± SEM).

**S. Table 4. Summary of statistical analyses of evaluation data of audio recordings in Experiment 2.**

|  |  | **Emotional valence** | | **Arousal** | |
| --- | --- | --- | --- | --- | --- |
|  |  | ***t*** | ***p*** | ***t*** | ***p*** |
| **Positive** | **Social** | 1.60 | 0.128 | -0.04 | 0.967 |
|  | **Nonsocial** |  |  |  |  |
| **Neutral** | **Social** | -1.51 | 0.149 | -1.00 | 0.327 |
|  | **Nonsocial** |  |  |  |  |
| **Negative** | **Social** | -1.52 | 0.145 | -0.54 | 0.594 |
|  | **Nonsocial** |  |  |  |  |

**Note:** Total stimulus material of sounds evaluation data by *t*-test. *p*-values associated with a single sample t-test on the differences of valence and arousal between social or nonsocial pictures*.* Stimuli were assessed by 40 participants who are not in the formal experiment.

**S. Results (**Detailed interaction effects in Experiment 1 & 2**)**

**Experiment 1**

***Behavioral data***

RTs were also modulated by the interaction of “emotion” × “sociality” (*F_2,57_* = 59.89, *p* < 0.001, η_p_^2^ = 0.51). Simple effects analyses indicated that, for the positive pictures, RTs to the social pictures were shorter than to the nonsocial pictures (social pictures: 893.54 ± 20.11 ms, nonsocial pictures: 992.06 ± 27.64 ms; *F_2,57_* = 24.98, *p* < 0.001, η_p_^2^ = 0.30), whereas for negative pictures, RTs to the social pictures (1005.05 ± 24.06 ms) were longer than to the nonsocial pictures (853.92 ± 20.96 ms, *F_2,57_* = 173.45, *p* < 0.001, η_p_^2^ = 0.75).

ACCs were modulated by the interaction of “emotion” × “sociality” (*F_2,57_* = 83.04, *p* < 0.001, η_p_^2^ = 0.58). Simple effects analyses indicated ACCs to nonsocial-positive pictures (65.5 ± 3.5 %) were lower than social-positive pictures (96.6 ± 0.7 %, *F_2,57_* = 86.15, *p* < 0.001, η_p_^2^= 0.60), whereas ACCs to nonsocial-negative (93.6 ± 0.8 %) and nonsocial-neutral (77.3 ± 2.0 %) pictures were higher than corresponding social-negative (89.4 ± 1.6 %, *F_2,57_* = 10.32, *p* = 0.002, η_p_^2^ = 0.15) and social-neutral (60.1 ± 2.5 %, *F_2,57_* = 48.90, *p* < 0.001, η_p_^2^ = 0.46) pictures.

The participants’ emotional responses were modulated by the interaction of “sociality” × “group” (*F_1,58_* = 7.94, *p =* 0.007, η_p_^2^ = 0.12). Simple effects analyses indicated that the emotional responses’ differences between Low-AQ and High-AQ groups were larger for social pictures (High-AQ group: 4.93 ± 0.13, Low-AQ group: 4.28 ± 0.12; *F_1,58_* = 13.09, *p =* 0.001, η_p_^2^ = 0.18) than for nonsocial pictures (High-AQ group: 4.36 ± 0.14, Low-AQ group: 4.55 ± 0.13; *F_1,58_* = 0.95, *p =* 0.333, η_p_^2^ = 0.02).

***ERPs data***

**P2**

The P2 amplitudes were significantly modulated by the interaction of “emotion” × “sociality” × “group” (*F_2,57_* = 3.95, *p* = 0.023, η_p_^2^= 0.06). Simple effects analyses indicated that for negative pictures, P2 amplitudes of social-negative pictures were smaller than nonsocial-negative pictures in Low AQ group (Social-negative: 5.66 ± 0.94 μV, nonsocial-negative: 6.88 ± 0.96 μV; *F_2,57_* = 10.30, *p* = 0.002, η_p_^2^= 0.15), but not in the High AQ group (Social-negative: 7.32 ± 0.93 μV, nonsocial-negative: 7.75 ± 0.96, *F_2,57_* = 1.25, *p* = 0.269, η_p_^2^= 0.02).

**P3**

The P3 amplitudes were significantly modulated by the interactions of “emotion” × “sociality” (*F_2,57_* = 4.31, *p* = 0.018, η_p_^2^ = 0.07). Simple effects analyses indicated that for social pictures, P3 amplitudes were significantly larger for social-positive pictures (8.85 ± 0.65 μV) than for social-neutral pictures (7.31 ± 0.74 μV, *F_2,57_* = 6.95, *p* = 0.002, η_p_^2^= 0.20). However, for nonsocial pictures, P3 amplitudes were not difference between nonsocial-positive (7.30 ± 0.59 μV) and nonsocial-neutral (7.26 ± 0.70 μV, *F_2,57_* = 0.16, *p* = 0.924, η_p_^2^ = 0.001) pictures. In addition, the P3 amplitudes were significantly modulated by the interaction of “emotion” × “sociality”× “group” (*F_2,57_* = 3.89, *p* = 0.026, η_p_^2^ = 0.06). Simple effects analyses indicated that P3 amplitudes in response to social-negative pictures were smaller than social-positive pictures in Low AQ group (Social-positive: 8.80 ± 0.92 μV, Social-negative: 6.54 ± 0.98 μV, *F_2,57_* = 9.53, *p* < 0.001, η_p_^2^ = 0.24), but not significant in the High AQ group (Social-positive: 8.91 ± 0.92 μV, Social-negative: 8.80 ± 0.92 μV; *F_2,57_* = 0.54, *p* = 0.536, η_p_^2^ = 0.18). No other main effect or interaction was found (*p* > 0.05 for all comparisons).

**LPP**

The LPP amplitudes were significantly modulated by the main effects of “sociality” (*F_1,58_* = 4.34, *p* = 0.042, η_p_^2^ = 0.07) and “emotion” (*F_2,57_* = 12.22, *p* = 0.001, η_p_^2^ = 0.17). Social pictures elicited larger LPP amplitudes than nonsocial pictures (Social: 6.77 ± 0.72 μV, nonsocial: 6.18 ± 0.69). Post hoc comparisons showed positive pictures (7.51 ± 0.59 μV) elicited larger LPP amplitudes than negative (6.33 ± 0.76 μV, *p* = 0.003) and neutral pictures (5.69 ± 0.78 μV, *p* < 0.001). However, no significant LPP amplitudes differences were observed between the negative and neutral pictures (*p* = 0.064).

The LPP amplitudes were significantly modulated by the interactions of “emotion” × “sociality” (*F_2,57_* = 6.34, *p* = 0.003, η_p_^2^ = 0.10). Simple effects analyses indicated that for social pictures, LPP amplitudes were significantly larger to social-positive pictures (8.45 ± 0.72 μV) than neutral pictures (5.53 ± 0.87 μV, *F_2,57_* = 12.54, *p* < 0.001, η_p_^2^ = 0.31), whereas, for nonsocial pictures, P3 amplitudes not different between nonsocial-positive pictures (6.35 ± 0.66 μV) and nonsocial-neutral pictures (5.84 ± 0.87 μV, *F_2,57_* = 0.75, *p* = 0.475, η_p_^2^ = 0.03). Importantly, the LPP amplitudes were modulated by the interaction of “emotion” × “sociality” × “group” (*F_2,57_* = 3.15, *p* = 0.047, η_p_^2^=0.05). Simple effects analyses indicated that, for social-negative pictures, LPP amplitudes were smaller in High-AQ group than Low-AQ group (High-AQ group: 4.66 ± 1.07 μV, Low-AQ group: 7.98 ± 1.06 μV; *F_2_*_,62_ = 4.83, *p* = 0.032, η_p_^2^ =0.08). There was no difference in the LPP amplitudes between groups in other conditions (*p* > 0.05 for all comparisons).

**Experiment 2**

***ERPs data***

**N1**

The N1 amplitudes were significantly modulated by the main effects of “sociality” (*F_1,63_* = 37.21, *p* < 0.001, η_p_^2^ = 0.37) and “group” (*F_1,63_* =7.92, *p* = 0.007, η_p_^2^= 0.11), social audio recordings elicited larger N1 amplitudes than nonsocial audio recordings (Social: −3.50 ± 0.25 μV, nonsocial: −2.57 ± 0.22 μV), and N1 amplitudes in Low-AQ group were larger than High-AQ group (Low-AQ group: −3.66 ± 0.32 μV, High-AQ group: −2.41 ± 0.31 μV).

The N1 amplitudes were significantly modulated by the interaction of “emotion” × “sociality” (*F_2,62_* = 108.13, *p* < 0.001, η_p_^2^ = 0.63). Simple effects analyses indicated that for social audio recordings, N1 amplitudes were significantly smaller for social-neutral audio recordings (−2.44 ± 0.26 μV) than for social-positive audio recordings (−4.68 ± 0.30 μV, *F_2,62_* = 60.51, *p* < 0.001, η_p_^2^ = 0.66). But, for nonsocial audio recordings, N1 amplitudes were significantly larger for nonsocial-neutral audio recordings (−3.71 ± 0.29 μV) than for nonsocial-positive audio recordings (−1.48 ± 0.23 μV, *F_2,62_* = 58.81, *p* < 0.001, η_p_^2^= 0.66). No significant main effect or interaction between the High-AQ and Low-AQ groups were observed in the N1 amplitude (*p* > 0.05 for all comparisons).

**P2**

The P2 amplitudes were significantly modulated by the main effect of “sociality” (*F_1,63_* = 29.95, *p* < 0.001, η_p_^2^ = 0.32) with social audio recordings elicited larger P2 amplitudes than nonsocial audio recordings (Social: 4.22 ± 0.30 μV, nonsocial: 3.14 ± 0.27 μV). The P2 amplitudes were significantly modulated by the interaction of “emotion” × “sociality” (*F_2,62_* = 26.82, *p* < 0.001, η_p_^2^ = 0.46), simple effects analyses indicated that for social audio recordings, P2 amplitudes were significantly smaller for social-neutral audio recordings (4.13 ± 0.31 μV) than for social-positive sounds (4.90 ± 0.35 μV, *F_2,62_* = 8.83, *p* < 0.001, η_p_^2^ = 0.22). But, for nonsocial audio recordings, N1 amplitudes were significantly larger for nonsocial-neutral audio recordings (3.25 ± 0.34 μV) than for nonsocial-positive audio recordings (2.55 ± 0.24 μV, *F_2,62_* = 5.69, *p* = 0.020, η_p_^2^ = 0.82). No significant main effect or interaction between the High-AQ and Low-AQ groups were observed in the P2 amplitudes (*p* > 0.05 for all comparisons).

**LNC**

The LNC amplitudes were significantly modulated by the main effect of “sociality” (*F_1,63_* = 13.60, *p* < 0.001, η_p_^2^ = 0.18) with social audio recordings elicited larger LNC amplitudes than nonsocial audio recordings (Social: −2.92 ± 0.23 μV, nonsocial: −2.31 ± 0.18). The LNC amplitudes were significantly modulated by the interaction of “emotion” × “sociality” (*F_2,62_* = 31.41, *p* < 0.001, η_p_^2^= 0.33). Simple effects analyses indicated that for social audio recordings, LNC amplitudes were significantly smaller for social-neutral audio recordings (−2.16 ± 0.26 μV) than for social-positive audio recordings (−3.06 ± 0.29 μV, *F_2,62_* = 13.96, *p* < 0.001, η_p_^2^= 0.18) and social-negative audio recordings ( −3.52 ± 0.25 μV, *F_2,62_* = 43.64, *p* < 0.001, η_p_^2^= 0.41). But, for nonsocial audio recordings, LNC amplitudes were significantly larger for nonsocial-neutral audio recordings (−2.89 ± 0.22 μV) than for nonsocial-positive audio recordings (−2.14 ± 0.24 μV, *F_2,62_* = 13.65, *p* < 0.001, η_p_^2^ = 0.18) and nonsocial-negative audio recordings (−1.85 ± 0.20 μV, *F_2,62_* = 27.02, *p* < 0.001, η_p_^2^ = 0.30).

Importantly, the LNC amplitudes were significantly modulated by the interaction of “emotion” × “sociality” × “group” (*F_2,62_* = 3.86, *p* = 0.025, η_p_^2^ = 0.06). Simple effects analyses indicated that, for social-negative audio recordings, LNC amplitudes were significantly smaller for High-AQ group than Low-AQ group (Low-AQ group: −4.12 ± 0.35 μV, High-AQ group: −2.93 ± 0.34 μV; *F_2_*_,62_ = 5.77, *p* = 0.019, η_p_^2^ = 0.08). However, there was no difference in the LNC amplitudes between groups in other conditions (*p* > 0.05 for all comparisons).
